# The Role of Propionibacterium acnes in the Pathogenesis of Sarcoidosis and Ulcerative Colitis: How This Connection May Inspire Novel Management of These Conditions

**DOI:** 10.7759/cureus.10812

**Published:** 2020-10-05

**Authors:** Sirisha Sakhamuru, Srikala Kambampati, Shehnaz Wasim, Vishal Kukkar, Bilal Haider Malik

**Affiliations:** 1 Internal Medicine, California Institute of Behavioral Neurosciences & Psychology, Fairfield, USA; 2 Radiology, California Institute of Behavioral Neurosciences & Psychology, Fairfield, USA

**Keywords:** propionibacterium acnes, sarcoidosis, ulcerative colitis, isotretinoin, noncaseating granuloma, cutibacterium acnes, inflammatory bowel disease, hidradenitis suppurativa, innate immune system, toll-like receptors

## Abstract

A lesser-acknowledged role of Propionibacterium acnes is its effect on the development of sarcoidosis. This literature review not only further explores this association but also that of Propionibacterium acnes and other inflammatory conditions, such as ulcerative colitis and pyoderma gangrenosum, acne, ulcerative colitis syndrome (PAC syndrome). This article reviews the effect that isotretinoin, a commonly used treatment of acne, has on the pathogenesis of ulcerative colitis, and the immune dysregulation and genetic susceptibility of individuals prone to developing acne, sarcoidosis, and ulcerative colitis.

Literature for this article review was obtained from PubMed by utilizing both regular keywords and medical subject heading (MeSH) subheadings for data gathering. Regular keywords were: Propionibacterium acnes, sarcoidosis, ulcerative colitis, and isotretinoin. MeSH subheadings used were: Propionibacterium acnes/immunology, Propionibacterium acnes/pathogenicity, Propionibacterium acnes/genetics, sarcoidosis/immunology, and sarcoidosis/genetics.

Following the application of inclusion and exclusion criteria, a total of 5172 publications were obtained. A total of 5086 publications were removed due to a lack of relevancy to outcomes of interest. The remaining 86 publications from all the regular and MeSH keywords were selected due to relevancy to outcomes of interest. Following this, a refined manual search was done, with the removal of duplicates, and 33 publications from PubMed were selected for review.

Following a review of these records, Propionibacterium acnes was repeatedly concluded to be a causative agent of sarcoidosis. Variable results for the association between Propionibacterium acnes and ulcerative colitis were found. Most studies showed no significant association between the use of isotretinoin and the development of ulcerative colitis. A strong overlapping role of genetic susceptibility and immune dysregulation in the pathogeneses of sarcoidosis, ulcerative colitis, and Propionibacterium acnes was found.

## Introduction and background

**Figure 1:**
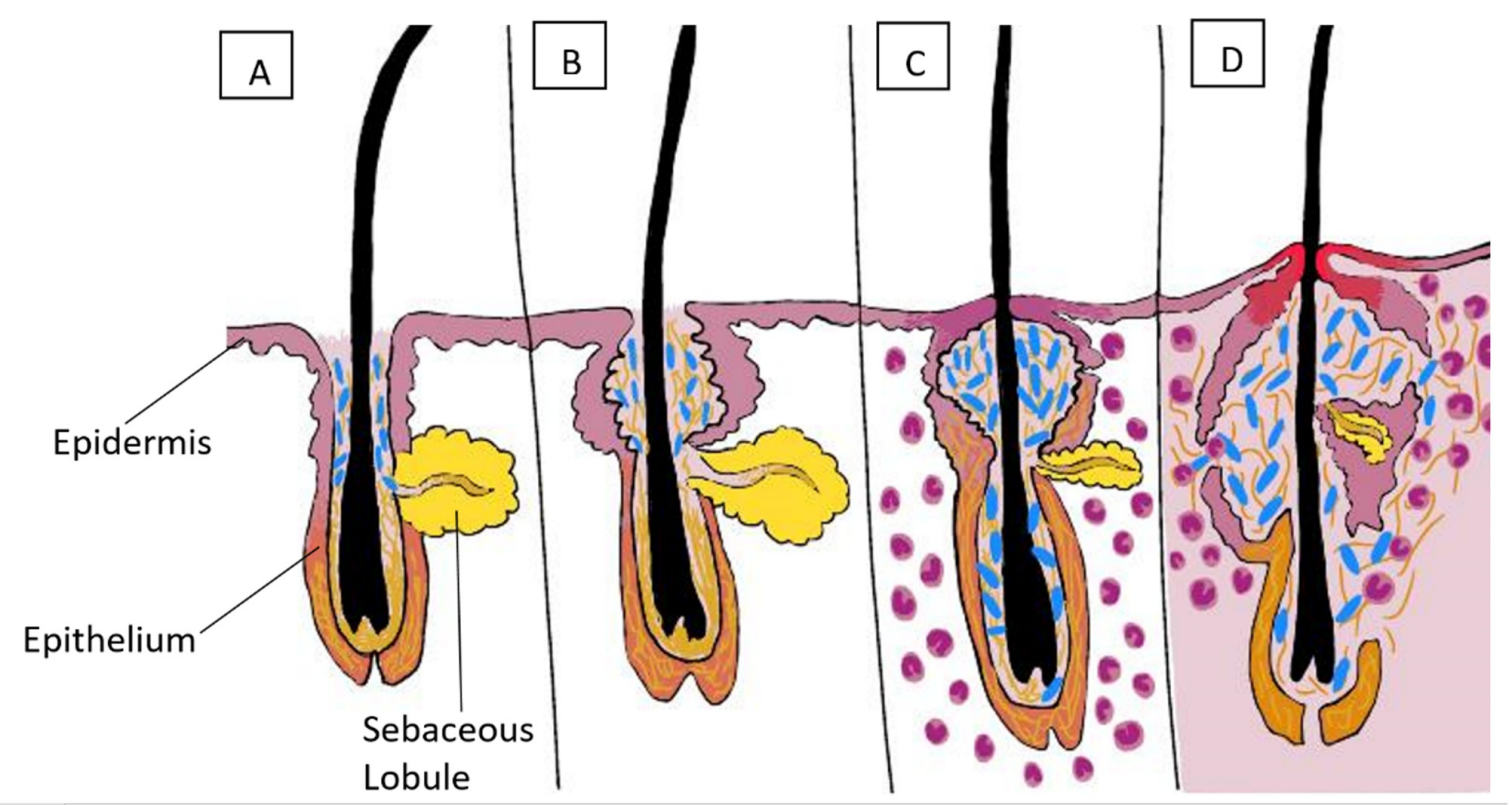
The pathogenesis of acne (A): Early comedone showing hyperkeratosis and androgens stimulating sebum secretion. (B): Later comedone with accumulated shed keratin and sebum. (C): Propionibacterium acnes proliferation (in blue) and sebaceous lobule regression. Mild inflammation. (D): Nodulocystic-marked inflammation and scarring.

Propionibacterium acnes (P. acnes) is a gram-positive bacterium, found ubiquitously as a commensal on the surface of the skin, the bowel, the conjunctival surface, the oral mucosa, and even the external ear canal. Perhaps the most well-known disease association with P. acnes is its role in the development of acne. This bacterium thrives in an anaerobic environment and has a predilection for the pilosebaceous unit of the skin, leading to epidermal inflammation and scarring, as seen in Figure 1. However, a lesser-acknowledged association of P. acnes is the role that it may play in the pathogenesis of sarcoidosis and ulcerative colitis.

Sarcoidosis is a systemic granulomatous inflammatory condition characterized by widespread noncaseating granulomas, which are most frequently formed in the skin, eyes, lymphatic system, and the lungs. Depending on the ethnicity of the population, the annual incidence of sarcoidosis varies significantly; the highest incidence is seen among African Americans (17-35 per 100,000 population), followed by Caucasians (5-12 per 100,000 population), and the lowest incidence is reported in the Asian and Hispanic populations (1-3 per 100,000 population) [1].

**Figure 2:**
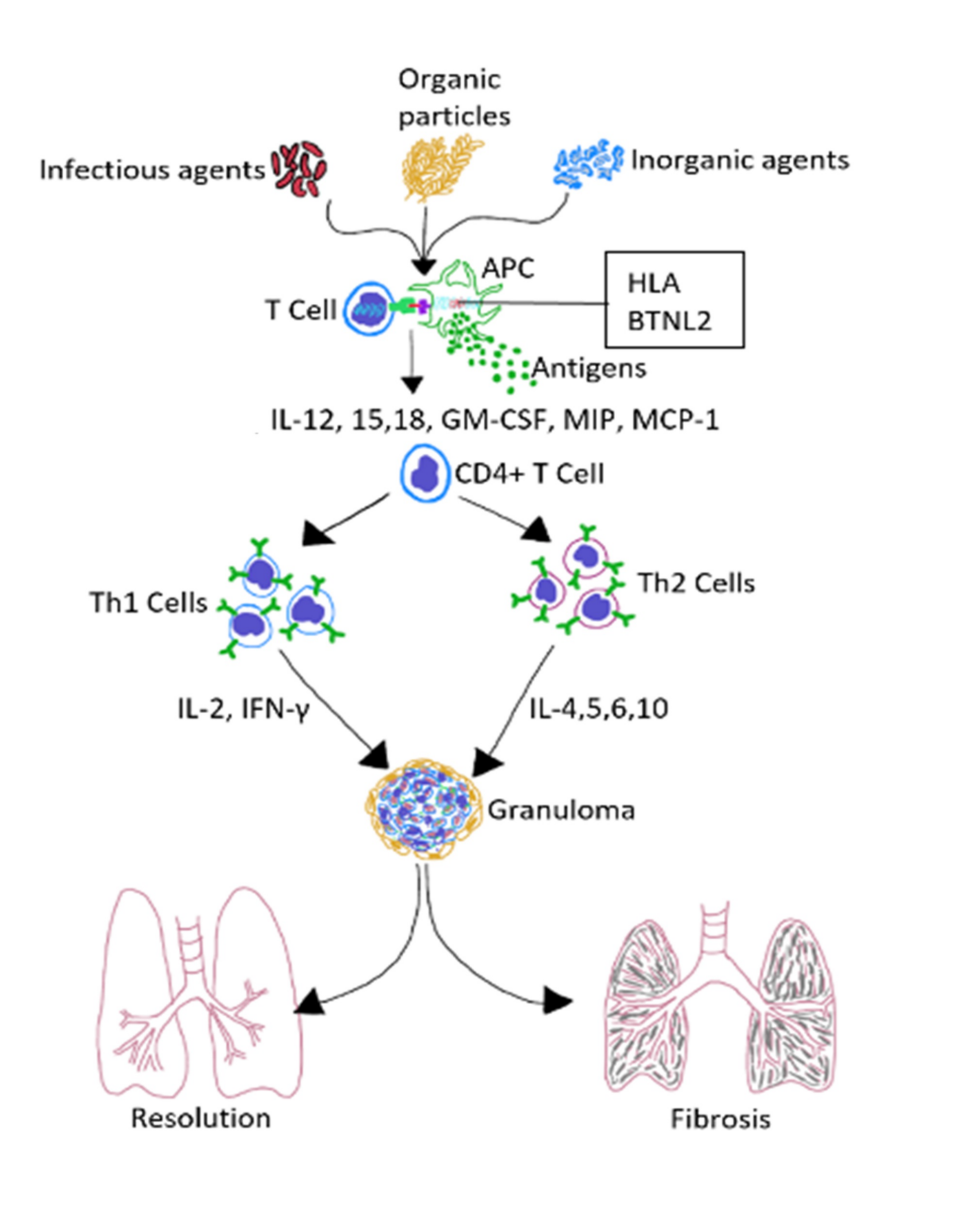
Hypothesized immunopathogenesis of sarcoidosis APC: antigen-presenting cell, HLA: human leukocyte antigen, BTNL2: butyrophilin-like 2, IL: interleukin, GM-CSF: granulocyte-macrophage colony-stimulating factor, MIP: macrophage inflammatory protein, MCP-1: monocyte chemoattractant protein-1, CD4+ T cell: cluster of differentiation 4 T cell, Th: helper T, INF-γ: interferon-gamma

Possible triggers of sarcoidosis include infectious agents, organic particles, and inorganic environmental factors, as shown in Figure 2. Some hypothesized infectious agents are P. acnes, mycobacteria, and human herpesvirus 8 [2]. Although the inciting agent is initially eradicated from the body, there are often non-degradable products left behind, which can induce immune responses that cross-react with self-antigens. Cluster of differentiation 4 T cells (CD4+ T cells) interact with antigen-presenting cells (APCs) to elicit the formation of noncaseating granulomas. The granulomas wall-off the non-degradable products left behind and either persist, resolve, or lead to fibrosis. There have been studies suggesting that individuals with genetically increased susceptibility to P. acnes infection also have an increased incidence of granuloma formation and sarcoidosis development. This review will further delve into the factors at the cellular level, such as the influence of the toll-like receptor (TLR) genes and the downstream signaling molecule myeloid-differentiation-primary-response-88 (MyD88) [3], to name a few, in the susceptibility to P. acnes, and therefore eventual granuloma development in sarcoidosis.

**Figure 3:**
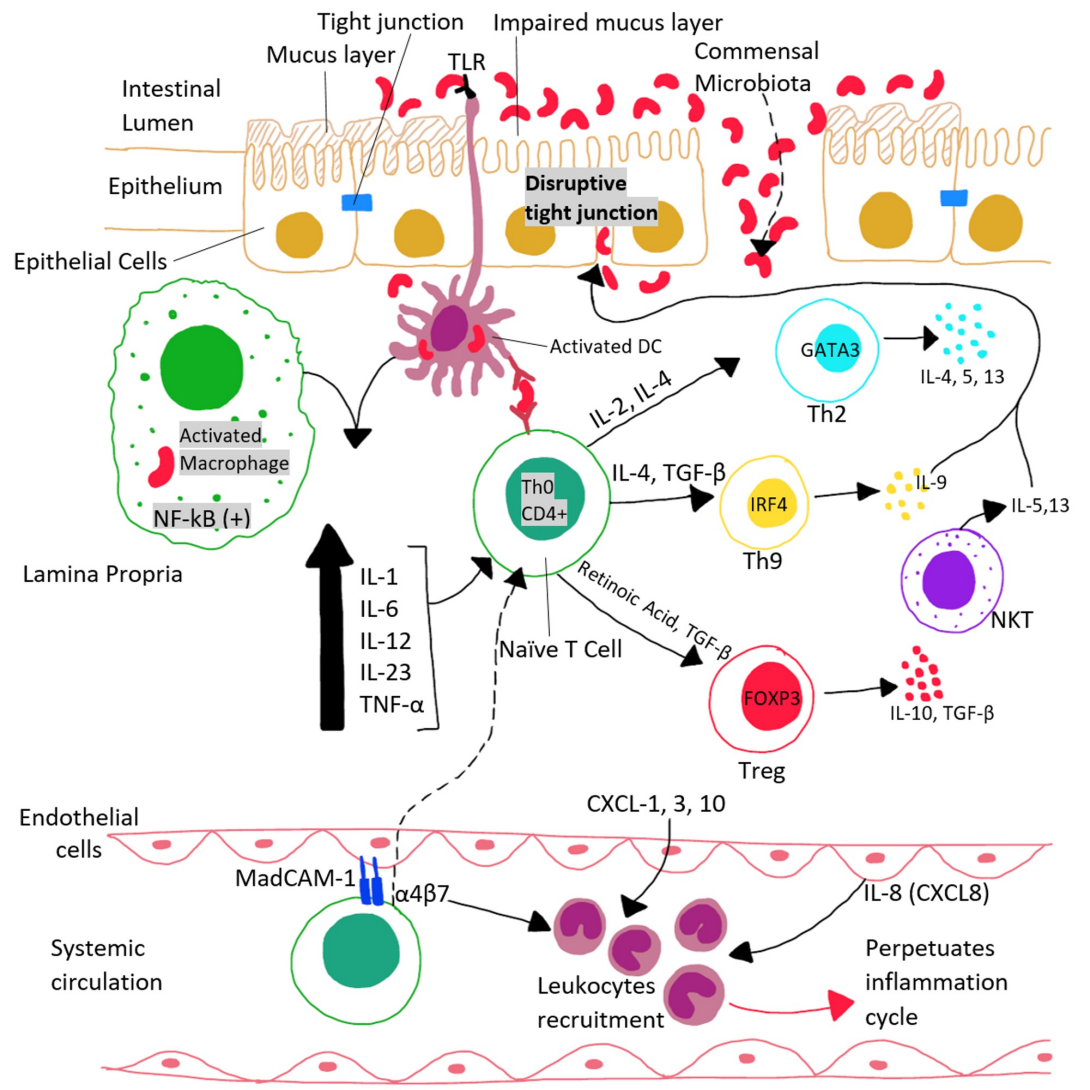
The pathogenesis of ulcerative colitis TLR: toll-like receptor, NF-kB: nuclear factor kappa-light-chain-enhancer of activated B cells, IL: interleukin, TNF-α: tumor necrosis factor-alpha, Th: helper T, CD4+: cluster of differentiation 4, TGF-β: transforming growth factor-beta, GATA3: transcription factor encoded by the GATA3 gene, IRF-4: interferon regulatory factor-4, NKT: natural killer T-cells, Treg: regulatory T cells, FOXP3: forkhead box P3, CXCL: chemokine (C-X-C motif) ligand, MadCAM-1: mucosal vascular addressin cell adhesion molecule-1, α4β7: alpha-4 beta-7 integrin protein

Like sarcoidosis, ulcerative colitis (UC) is also a multi-system condition, although it mainly involves the colon and rectum. As shown in Figure 3, changes in the commensal gut bacteria and impairment of the intestinal luminal mucosa and epithelium due to disruption of tight junctions are the main implicating factors in the pathogenesis of ulcerative colitis. Ulcerative colitis is characterized by relapsing and remitting episodes of bloody diarrhea, abdominal pain, and systemic symptoms. It is most commonly seen in young adults, but any age group can be affected. Ulcerative colitis has become far more common over the last decade, predominantly in developing countries, such as Asia, Latin America, and Eastern Europe. In contrast, however, the actual prevalence of ulcerative colitis is far less in the Asian populations (5.3 to 63.6 per 100,000 people) than in North America (37.5 to 238 per 100,000 people) [4].

In addition, a particularly interesting association that has been repeatedly hypothesized in current literature is that the use of isotretinoin in the treatment of P. acnes is associated with an increased incidence of ulcerative colitis. If significant, this association could make it necessary to avoid isotretinoin use in patients prone to developing ulcerative colitis and its associated autoimmune conditions, such as primary sclerosing cholangitis [5].

The types of acne implicated in the development of sarcoidosis and ulcerative colitis will also be reviewed in this article. More specifically, repeated inflammation of the apocrine glands, also known as hidradenitis suppurativa (HS), or ‘acne inversa’ has also been associated with systemic inflammatory syndromes [6,7]. All these inflammatory conditions of the skin, both of the pilosebaceous glands and the apocrine glands, have been associated with systemic conditions sarcoidosis and ulcerative colitis.

Both these conditions share quite a few similarities in their pathogenicity; they are both the result of cellular immune dysregulation [8,9]. This is precisely where the organism P. acnes comes into play. If it is found that this organism is significantly implicated in the pathogenesis of these diseases, we can focus our management of the conditions on reducing the commensal load of P. acnes in affected patients. This article aims to review the role of P. acnes in the development of sarcoidosis and ulcerative colitis and to establish if there is a commonality between the immune dysregulation present in both conditions.

## Review

Method

Literature for this article review was obtained from PubMed by utilizing both regular keywords and medical subject heading (MeSH) subheadings for data gathering. Table 1 shows the regular and MeSH keywords searched for during the collection of data.

**Table 1:** The regular and MeSH keywords used MeSH: medical subject headings ^1^Duplicate studies were removed from this list, and the resulting 33 studies were added to the datasheet.

Regular Keywords	Total Records	After Applying Inclusion/Exclusion Criteria	Records Selected for Datasheet^1^
Propionibacterium acnes	5178	208	15
Sarcoidosis	31088	1277	21
Ulcerative Colitis	49251	3305	21
Isotretinoin	4615	156	7
MeSH Keywords	Total Records	After Applying Inclusion/Exclusion Criteria	Records Selected for Datasheet^1^
Propionibacterium acnes/immunology	1369	22	6
Propionibacterium acnes/pathogenicity	192	20	3
Propionibacterium acnes/genetics	190	21	3
Sarcoidosis/immunology	2382	89	7
Sarcoidosis/genetics	822	74	3

Inclusion Criteria

All records published in the last 10 years, in the English language, which had the availability of the abstract, free full texts, and full text, were included. All records consisting of the following article types were included: case reports, classical article, clinical conference, clinical study, clinical trial, clinical trial protocol, controlled clinical trial, journal article, meta-analysis, multicenter study, newspaper article, observational study, randomized controlled trial, review, and systematic review. The species included humans and other animals. The journal category included was MEDLINE.

Exclusion Criteria

All records published more than 10 years ago or not in the English language were excluded. All records consisting of the following article types were excluded: address, autobiography, bibliography, biography, books and documents, clinical trial-phase I, clinical trial-phase II, clinical trial-phase III, clinical trial-phase IV, clinical trial-veterinary, comment, comparative study, congress, consensus development conference, consensus development conference, NIH, corrected and republished article, dataset, dictionary, directory, duplicate publication, editorial, electronic supplementary materials, English abstract, evaluation study, government publication, guideline, historical article, interactive tutorial, interview, introductory journal article, lecture, legal case, legislation, letter, and news. The journal categories excluded were dental journals and nursing journals.

Results

Table 2 shows the total number of articles after applying the inclusion/exclusion criteria.

**Table 2:** Total records obtained following application of inclusion/exclusion criteria MeSH: medical subject headings ^1^Text availability: abstract, free full texts, full text ^2^Article types included: case reports, classical articles, clinical conferences, clinical study, clinical trials, clinical trial protocol, controlled clinical trial, journal article, meta-analysis, multicenter study, newspaper article, observational study, randomized controlled trial, review, and systematic review Article types excluded: address, autobiography, bibliography, biography, books and documents, clinical trial-phase I, clinical trial-phase II, clinical trial-phase III, clinical trial-phase IV, clinical trial-veterinary, comment, comparative study, congress, consensus development conference, consensus development conference, NIH, corrected and republished article, dataset, dictionary, directory, duplicate publication, editorial, electronic supplementary materials, English abstract, evaluation study, government publication, guideline, historical article, interactive tutorial, interview, introductory journal article, lecture, legal case, legislation, letter, and news

	Regular Keyword - Propionibacterium acnes
Total Records	5178
Inclusion/Exclusion:	
Published within 10 years	1656
Language - English	1613
Text Availability^1^	668
Article Types^2^	665
Journal Category - MEDLINE	438
Humans, Other Animals	400
Male, Female	208
	Regular Keyword - Sarcoidosis
Total Records	31088
Inclusion/Exclusion:	
Published within 10 years	8476
Language - English	7668
Text Availability^1^	2960
Article Types^2^	2948
Journal Category - MEDLINE	1632
Humans, Other Animals	1629
Male, Female	1277
	Regular Keyword - Ulcerative Colitis
Total Records	49251
Inclusion/Exclusion:	
Published within 10 years	19837
Language - English	18886
Text Availability^1^	7978
Article Types^2^	7928
Journal Category - MEDLINE	5105
Humans, Other Animals	5089
Male, Female	3305
	Regular Keyword - Isotretinoin
Total Records	4615
Inclusion/Exclusion:	
Published within 10 years	1506
Language - English	1420
Text Availability^1^	432
Article Types^2^	430
Journal Category - MEDLINE	234
Humans, Other Animals	232
Male, Female	156
	MeSH Keywords - Propionibacterium acnes/Immunology
Total Records	1369
Inclusion/Exclusion:	
Published within 10 years	92
Language - English	89
Text Availability^1^	44
Article Types^2^	44
Journal Category - MEDLINE	44
Humans, Other Animals	43
Male, Female	22
	MeSH Keywords - Propionibacterium acnes/pathogenicity
Total Records	192
Inclusion/Exclusion:	
Published within 10 years	86
Language - English	84
Text Availability^1^	34
Article Types^2^	34
Journal Category - MEDLINE	34
Humans, Other Animals	32
Male, Female	20
	MeSH Keywords - Propionibacterium acnes/genetics
Total Records	190
Inclusion/Exclusion:	
Published within 10 years	124
Language - English	120
Text Availability^1^	60
Article Types^2^	60
Journal Category - MEDLINE	60
Humans, Other Animals	54
Male, Female	21
	MeSH Keywords - Sarcoidosis/immunology
Total Records	2382
Inclusion/Exclusion:	
Published within 10 years	358
Language - English	339
Text Availability^1^	123
Article Types^2^	123
Journal Category - MEDLINE	123
Humans, Other Animals	123
Male, Female	89
	MeSH Keywords - Sarcoidosis/genetics
Total Records	822
Inclusion/Exclusion:	
Published within 10 years	253
Language - English	239
Text Availability^1^	115
Article Types^2^	113
Journal Category - MEDLINE	113
Humans, Other Animals	113
Male, Female	74

**Figure 4:**
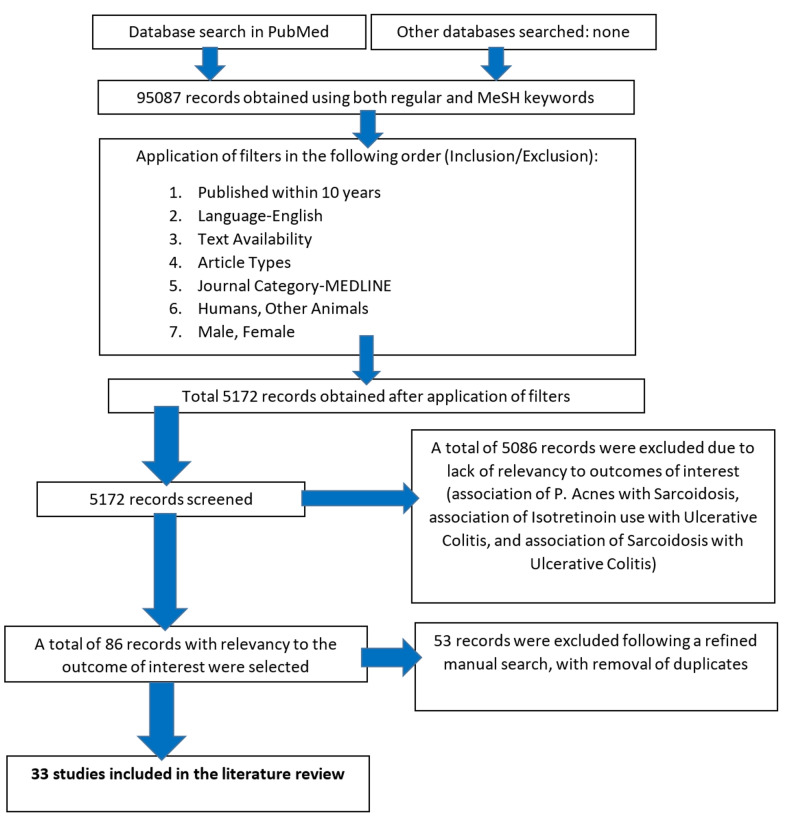
The data collection process MeSH: medical subject headings P. acnes: Propionibacterium acnes

For both the regular and MeSH keywords, following the application of inclusion/exclusion criteria in PubMed, a total of 5172 publications were obtained. A total of 5086 publications were removed due to lack of relevancy to outcomes of interest: association of P. acnes with sarcoidosis, association of isotretinoin use (P. acnes treatment) with ulcerative colitis, and association of sarcoidosis with ulcerative colitis. The remaining 86 publications from all the regular and MeSH keywords were selected due to relevancy to outcomes of interest. Following this, a refined manual search was done, with the removal of duplicates, and 33 publications from PubMed were selected for review as follows: twelve case-control studies, five article reviews, three observational studies, four animal studies, three case studies, two cohort studies, two case reports, and two human studies. All the articles reviewed are available for reference. A more in-depth review was performed for the available publications after inclusion/exclusion to include the relevant topic of interest and its significant associations. Figure 4 shows the process of collection of records for the current literature review.

Discussion

The Role of Genetics and the Immune System in the Development of P. acnes Infection

Many factors contribute to the development of P. acnes infection, and it is evident that the interaction between P. acnes and host immunity plays a significant role in this. The ubiquitous nature of P. acnes was previously thought to be due to the overproduction of the organism in all areas with high sebum production. However, more recent studies, through genomic sequencing of the P. acnes organism, have shown that specific individuals may be more predisposed to developing P. acnes infection. The genes most commonly involved in the pathogenesis of P. acnes are those that code for components of the innate immune system, such as TLRs, interleukins (ILs), and other inflammatory signaling pathways in the host.

P. acnes aggravates acne by promoting the synthesis of lipids, the formation of comedones, and by inducing and precipitating inflammation in the host's skin. The inflammatory action of P. acnes occurs through the activation of TLR-2 and TLR-4 on keratinocytes, which in turn activates the mitogen-activated protein kinase (MAPK) and nuclear factor kappa-light-chain-enhancer of activated B cell (NF-kB) pathways of inflammation. NF-kB is a transcriptional factor that is responsible for the regulation of gene expression in the inflammatory cascade. This demonstrates the ability of P. acnes to selectively activate certain innate immunity genes [10]. These inflammatory pathways, as a result, stimulate the production of IL-1, IL-6, and IL-8, tumor necrosis factor-α, and granulocyte-macrophage colony-stimulating factor (GM-CSF) from keratinocytes [9].

Commonly thought to be found only in patients suffering from acne vulgaris, P. acnes can also be found in healthy subjects who are not suffering from acne. Genomic studies of P. acnes have revealed that varying strains are associated with different degrees of acne. Strains RT4/5 are more strongly associated with acne, while strains RT2/6 are associated with healthy human skin. Specific pathogenic factors encoded in the acne-inducing strains were identified in genomic studies of P. acnes. In a murine model of acne, done by inoculating P. acnes with a synthetically produced human sebum, very little loss of viability was observed after one week (indicating intracellular persistence of P. acnes). The acne-associated P. acnes strains (RT4/5), compared with the health-associated P. acnes strains (RT2/6), induced significantly higher levels of IL-8, IL-1α, IL-1β, and IL-6 in vitro and in vivo [11]. This study shows that the genomic factors of P. acnes need to be further evaluated in order to possibly develop new approaches to managing acne.

P. acnes can induce particular genetic expression at the cellular level, such as increased expression of the genes coding for caspase-1 and inflammasomes in monocytes, subsequently causing the release of IL-1β from these cells. Through this mechanism, P. acnes triggers mononuclear cells to release IL-6, IL-1β, and transforming growth factor-β (TGF-β) and stimulates the differentiation of CD4+ T lymphocytes into helper T cells 1 and 17 (Th1 and Th17) cells. These derived cells are responsible for the upregulation of IL-17 and that of interferon-gamma [9].

In an observational study done to better understand the body’s adaptive immune responses to P. acnes, it was shown that infiltrating immune cells consisted mostly of CD4+ T cells in the area surrounding the follicles affected by P. acnes. By studying specific CD4+ T cells in vitro, the results of this study showed that P. acnes induces a mixed helper T cell (Th17/Th1) response by inducing the secretion of IL-17A and interferon-gamma from these cells [12].

Interferon-gamma is a cytokine best known for its role in the activation of macrophages and the synthesis and maintenance of granulomas, which are the pathognomic findings of sarcoidosis. In a human study of a macrophage cell line, the intracellular persistence of P. acnes was demonstrated. The P. acnes-containing phagosome did not fuse with lysosomes, preventing the formation of the phagolysosome, and therefore allowing P. acnes to persist intracellularly [13]. This mechanism of defective phagolysosome production and the perseverance of infective organisms are also commonly implicated in the formation of granulomas.

How the Innate Immune System Plays a Role in the Pathogenesis of Sarcoidosis and Ulcerative Colitis

The development of sarcoidosis can be attributed to several factors involved in the innate immune system. In order to demonstrate this, an animal study was conducted where mice were injected with a viable strain of P. acnes isolated from a patient with sarcoidosis. The mice deficient in the innate immunity adapter proteins MyD88 and cytochrome B-245 beta chain (CybB) had larger and more granulomas compared to the wild-type mice injected with P. acnes. Typically, in response to P. acnes administration in wild-type mice, proinflammatory markers are synthesized by the body, and neutrophils are transported into the lung, a response that is dependent on MyD88 and CybB [6]. In the absence of this response, granulomas form as a result of the inability of the body to clear the pathogenic substance.

A case-control study was done to determine possible genetic and functional differences in TLR-9 between patients with sarcoidosis and healthy controls. To assess any genetic differences in these groups, the promoter region of the TLR-9 gene was sequenced. However, no genetic differences were found between the patients and controls. Additionally, in assessing the functional aspect of TLR-9, patients with sarcoidosis produced significantly less interferon-gamma upon stimulation with different stimuli. IL-23 and IL-6 productions were also measured. A significant difference between the case-control groups was not found here either, except when stimulated by a TLR-9 agonist, which increased IL-23 production. The study concluded that the decreased production of interferon-gamma could reflect the anergic state seen in the peripheral blood T lymphocytes of patients with sarcoidosis, and also concluded that the ability of TLR-9 to increase the production of IL-23 could indicate that functional defects in the immune pathway of TLR-9 could increase the susceptibility to sarcoidosis [14].

Moreover, reiterating these results about TLR-9, a randomized controlled trial was done to test the ability of TLRs to act as drug targets for immunomodulation in ulcerative colitis. The medication, DIMS0150 (a TLR-9 agonist), was given to patients, and the study compared a control group with a placebo. Significantly (p=0.0073) higher mucosal healing and histological improvement were seen in the TLR-9 agonist group after four weeks [15]. This study illustrates the promising new therapeutic options for patients with ulcerative colitis.

In another study involving TLRs, their role in the development of sarcoidosis was studied. Bronchoalveolar (BAL) lavage cells from patients with sarcoidosis and healthy controls were stimulated with ligands taken from TLR-2 and TLR-4. Results were measured based on the production of tumor necrosis factor-α and IL-6. Patients with sarcoidosis showed a significantly higher cytokine response than the controls [16]. A subsequent murine model study was done to further evaluate the impact of TLR-2 in Th1-associated lung disease induced by P. acnes. The number of granulomatous lesions in the lungs was markedly attenuated in TLR-2 gene deleted mice compared to the wild-type mice [16]. These studies show that there is a significant impact of inadequate TLR-2 responses on the development of sarcoidosis.

Let us consider the case report of a 30-year-old patient with ulcerative colitis, who was in remission and on maintenance Infliximab therapy. He was admitted with symptoms of dyspnea, nocturnal sweating and, nonproductive cough. Following further investigations, he was found to have drug-induced stage II pulmonary sarcoidosis. This report demonstrates the importance of monitoring patients under biological therapy using agents such as anti-tumor necrosis factor-α. Monitoring such patients can identify paradoxical adverse effects of inflammation, such as the development of sarcoidosis [17].

In addition to its role in the pathogenesis of sarcoidosis, the innate immune system also can be implicated in other inflammatory conditions, such as ulcerative colitis. One case-control study investigated whether immune-mediated diseases are more frequent in patients with inflammatory bowel disease (IBD). Results showed a significantly (p<0.05) greater percentage of comorbidity in the IBD groups than the controls. Odds ratios were significantly increased (p<0.00125) for primary sclerosing cholangitis, celiac disease, type 1 diabetes, sarcoidosis, asthma, iridocyclitis, psoriasis, pyoderma gangrenosum, rheumatoid arthritis, temporal arteritis, and atrophic gastritis [18].

In an animal study conducted to study the goblet cell dysfunction that underlies the pathology of ulcerative colitis, the role of the immune system in the pathogenesis of ulcerative colitis is exemplified. The mucosal barrier of the intestine is responsible for mediating the commensal bacteria in the gut. This mucosa is maintained by the goblet cells, which synthesize the protective mucus layer. When this layer is impaired, ulcerative colitis ensues. IL-18 is the main factor that can cause the barrier integrity in a model of colitis to be broken down. In fact, when the IL-18 or its receptor in the intestinal epithelial cells were deleted in mice, this resulted in the lack of ulcerative colitis development and a lack of mucosal damage [19].

The Role of P. acnes in the Development of Sarcoidosis

In assessing the role of P. acnes in the development of sarcoidosis, a case-control study was done. This study aimed to detect P. acnes-derived immune complexes in sarcoid lesions. Lymph node samples from patients with sarcoidosis and a P. acnes-specific monoclonal antibody were used to react with the bacterium P. acnes. Results showed that insoluble immune complexes (IICs)-forming P. acnes were present in 89% of the sarcoid samples, and only 20% of the control samples with lymphadenitis. In addition, no insoluble immune complexes were detected in control samples without lymphadenitis. Results also showed a high number of P. acnes-derived IICs in macrophages in sarcoid lymph nodes [20]. All these factors show there could be an etiological connection between P. acnes and sarcoidosis.

In another case-control study done to assess the link between P. acnes and sarcoidosis, the ability of P. acnes to induce granuloma formation in the lungs of patients was assessed. When stimulated with heat-killed P. acnes, the production of tumor necrosis factor-α and GM-CSF by BAL cells were measured. The BAL cells of patients with sarcoidosis induced significantly higher levels of the inflammatory cytokines when stimulated by P. acnes, as compared to the controls [21]. This further supports the hypothesis that P. acnes is one of the pathogens responsible for sarcoidosis.

An interesting case study, further supporting this association, showed that patients developed sarcoidosis six months following the use of etanercept (a tumor necrosis factor-inhibitor) for the treatment of rheumatoid arthritis. Here, immunohistochemistry revealed P. acnes in the noncaseating granulomas. This shows that even when Sarcoidosis is drug-induced, the primary pathogenic agent is commonly P. acnes [22].

Myocardial tissue from patients with known cardiac sarcoidosis, myocarditis, and other cardiomyopathies was obtained for an observational study. All three types of samples were formalin-fixed paraffin-embedded using immunohistochemistry with a P. acnes-specific monoclonal antibody. Results showed that P. acnes showed significantly high positive reactivity in the cardiac sarcoidosis group, with massive inflammation. In contrast, no reactivity was noted in the myocarditis and cardiomyopathy samples [23]. This repeated presence of P. acnes in sarcoid granulomas suggests that this commensal bacterium may be implicated in causing the granulomas in many patients with cardiac sarcoidosis. This will also help to differentiate between cardiac sarcoidosis and other cardiac pathologies.

A recent case-control study examined bacterial lymph node biopsy samples using 16SrRNA gene sequencing in patients with sarcoidosis to test the strength of association between P. acnes and sarcoidosis. P. acnes 16SrRNA was solely detected in all the samples of the sarcoidosis groups, and none was detected in the controls or tuberculosis (TB) groups. The quantitative amount of P. acnes bacterium found was also significantly (p=0.0010) higher than that amount found in tuberculosis samples [24]. These results further reiterate the involvement of P. acnes in patients who are diagnosed with sarcoidosis.

In one case-control study, the idea that P. acnes occurs more commonly in genetically susceptible individuals, and therefore causes sarcoidosis in these patients, was explored. Currently, the most common causative agents thought to cause sarcoidosis are both P. acnes and mycobacterium. However, on actual bacterial culture, only P. acnes is detected in the sarcoid lesions. In this case-control study, the P. acnes antibody was formalin-fixed and paraffin-embedded with both sarcoidosis and control samples. The immunohistochemistry showed that 74% of the lung samples reacted with the sarcoid samples. The antibody did not react with non-sarcoid granulomas or any of the 45 tuberculosis samples, strongly implicating P. acnes as the causative organism for the formation of granulomas in sarcoidosis [25].

Specific strains of Propionibacterium are implicated in sarcoidosis. In a previous study, complete genomic sequencing of a particular C1 strain of P. acnes was done, and this strain was the primary bacterial isolate from the granulomas of sarcoidosis cases. In a more recent observational study done, the genetic makeup of this C1 strain was further evaluated. The analysis showed that the C1 strain was phylogenetically unique and found primarily in the sarcoid isolate, identified by its unique 18.8-kilobase pairs (kbp) transposon sequence. Specific sequence types (STs) were identified, namely ST26 isolates, which carry the transposon insertion [26]. This study emphasizes the need to further study the ST26 P. acnes strain as a causative agent of sarcoidosis.

Some studies have gone a step further by evaluating the diagnostic value of using P. acnes rRNA as a biomarker for sarcoidosis. This potential biomarker was studied in a case-control study by using reverse-PCR to measure the amount of rRNA of P. acnes present in sarcoidosis and control cases. P. acnes rRNA was detected in 48 of the 65 sarcoidosis samples, but only four of the 45 tuberculosis samples and only three of the 50 controls. The number of copies determined as a cut-off from this study was 50.5 copies per milliliter. This study suggests the diagnostic ability to quantify P. acnes rRNA for distinguishing between sarcoidosis and tuberculosis [27].

The Role of P. acnes in the Development of Ulcerative Colitis

According to several existing studies, there is a recognized association between P. acnes and the gastrointestinal tract. It has been long hypothesized that the commensal bacteria in the gut could be causative in the pathogenesis of P. acnes infection. For example, when an individual is depressed or has anxiety, there has been evidence of exacerbation of acne due to the subsequent gut microbiome alteration and increased skin inflammation. There indeed is evidence of a gastrointestinal-dermal axis that interlinks the intestinal microbiota, oral commensal bacteria, and diet to acne. It has been suggested that the mechanism that allows the gut microbiome to influence skin homeostasis comes from its altering effects on innate immunity [9].

One pathway suggested to influence acne is the mechanistic target of rapamycin (mTOR) pathway when interacted with by intestinal flora. This pathway controls the intestinal barrier, and in the case of a disturbance of this barrier, a positive feedback loop that stimulates proinflammatory cytokines can be formed. As discussed earlier, this disturbance of the intestinal mucosal barrier by the organism P. acnes in the gut is the same mechanism by which ulcerative colitis can develop. Probiotics have been shown to directly inhibit P. acnes through their antimicrobial proteins [28].

In a cohort study done to evaluate the effect of isotretinoin on the development of ulcerative colitis, participants who were recently treated with isotretinoin or topical acne medications were studied. The result was no significant association between isotretinoin use and the development of IBD. However, in an additional prespecified secondary analysis, isotretinoin was associated with IBD in patients aged 12-19 years, and topical acne medications were also found to be associated with IBD. Since the primary study did not find a significant association in either group, but there was an association in prespecified secondary analyses, it was suggested that there is a possible association between IBD and P. acnes itself [29]. Similarly, in a 12-year observational study done to explore the association between isotretinoin and ulcerative colitis, it was concluded that P. acnes itself is more likely a causative agent in the development of ulcerative colitis [30]. Further studies and research are required to understand this association better.

Does Isotretinoin Use, for the Management of Acne, Increase the Risk of Developing Ulcerative Colitis?

Isotretinoin is a form of vitamin A commonly used to manage cases of severe acne vulgaris, such as nodulocystic acne. It has been hypothesized that this medication can trigger IBD, namely ulcerative colitis. A human study was conducted to review the effect of isotretinoin on the immune system. This study hypothesized that isotretinoin could cause acne to subside by normalizing the innate immune response to the commensal P. acnes. The blood monocytes of these patients with acne showed significantly high quantities of TLR-2 expression when stimulated by P. acnes. However, when treated with isotretinoin, this TLR-2 expression was significantly suppressed, and this effect lasted even six months following the therapy [31]. This demonstrates the ability of isotretinoin to modulate the immune system, namely TLR-2, and its possible influence on other inflammatory conditions affected by TLR-2 dysregulation.

The possible association between isotretinoin use and the development of ulcerative colitis was first documented in 1986 when a young woman developed proctitis during isotretinoin use. She reported relief of her bowel symptoms after withholding the isotretinoin medication. Since that time, several case-control and cohort studies have been done to study the possibility of this etiological link further. A case-control study done with a population of women using oral contraceptives concluded no association between the vitamin A analogue and ulcerative colitis (95% confidence interval=0.52-1.90) [5]. Another study, a retrospective population-based cohort, analyzed data from patients using isotretinoin, and similarly concluded that there was no connection between isotretinoin use and IBD [5].

Although the causal link is not proven to date, some studies do suggest some relationship between isotretinoin use and ulcerative colitis. A case-control study was done by following a population of IBD cases, all exposed to isotretinoin for a one-year duration. In this study, results were different from those of the cases discussed above. Ulcerative colitis was strongly associated with previous isotretinoin exposure (95% confidence interval of 1.97-9.66 with an odds ratio of 4.36). No association was found between the medication use and Crohn’s disease, or in the controls group. This study concluded that this association between ulcerative colitis and isotretinoin is dose-dependent, and the higher the dose, the more the risk. The absolute risk for developing ulcerative colitis following isotretinoin use is small. However, it is useful for clinicians to know this possible correlation when prescribing isotretinoin [32].

The Role of Genetic Susceptibility in Developing Hidradenitis Suppurativa With the Auto-Inflammatory PAC Syndrome

Hidradenitis suppurativa (HS), also known as ‘acne inversa’, is often associated with other auto-inflammatory conditions that can become severe if left undiagnosed or untreated. Hidradenitis suppurativa is caused by a dysfunction of the innate immune system, with abnormal IL-1 signaling that leads to repetitive inflammation by neutrophils at affected sites. This same abnormal pathway is shared with the other auto-inflammatory conditions such as acne, pyoderma gangrenosum, psoriatic arthritis, and ulcerative colitis [6]. When pyoderma gangrenosum, acne, and ulcerative colitis occur together, it is diagnosed as PAC syndrome. All these conditions share a similar pathogenesis, suggesting their overlapping etiologies.

A case study was done to evaluate the genetic susceptibility of patients who develop pyoderma gangrenosum, acne, and ulcerative colitis (PAC) syndrome. It is known that the gene PSTPIP1 is involved in immune regulation, and possibly the increased susceptibility to developing PAC syndrome. Considering this, a 33-year-old man with chronic ulcerative colitis, severe acne, and multiple non-healing ulcers in the lower limbs (PAC syndrome) was studied. The PSTPIP1 gene was sequenced and studied for any abnormalities. A heterozygous mutation was found that damaged the function of the resulting encoded protein, affecting the IL-1-signaling pathway. Following this, the human IL-1 antagonist anakinra was used to treat this patient, and his condition improved greatly. This study concluded that there is a commonality in the pathogenesis of pyoderma gangrenosum, acne, and ulcerative colitis, due to this mutation [7]. These PSTPIP1-associated diseases need to be further evaluated and possibly considered under one etiological cause.

An observational study evaluated a possible association between hidradenitis suppurativa and IBD. The prevalence and risk of IBD in patients with hidradenitis suppurativa compared to the general population were studied. It was concluded that the risk of both new-onset Crohn’s disease and ulcerative colitis was significantly increased in patients with hidradenitis suppurativa. However, the actual prevalence was still low [33]. These results emphasize the need for clinicians to assess gastrointestinal complaints in patients with hidradenitis suppurativa thoroughly and consider new-onset IBD as a possible cause.

## Conclusions

Although sarcoidosis has a multifactorial etiology, P. acnes has repeatedly been shown as a causative agent of sarcoidosis in several well-designed, reproducible studies. The link between P. acnes and ulcerative colitis, on the other hand, requires far more research because an association is suspected, but clinical studies have shown variable results. In addition, most studies showed no significant association between the use of isotretinoin and the development of ulcerative colitis. Based on the several studies reviewed, it can be established that there is indeed an overlapping role of genetic susceptibility and immune dysregulation in the pathogeneses of sarcoidosis, ulcerative colitis, and P. acnes. However, many of these results are obtained from animal studies, and further exploration in the form of human studies is required. This will help clinicians determine novel ways to direct their management of these conditions in the future by targeting these specific genetic and immune vulnerabilities.
